# qSOFA combined with suPAR for early risk detection and guidance of antibiotic treatment in emergency department: a bit sweet and a bit sour randomized controlled trial

**DOI:** 10.1186/s13054-024-04944-w

**Published:** 2024-05-13

**Authors:** Bharat Paliwal, Nikhil Kothari, Trishita Saha, Pradeep Bhatia

**Affiliations:** Department of Anaesthesiology and Critical Care, All India Institute of Medical Sciences, Jodhpur, Rajasthan India

With great interest, we read the article by Adami et al. [1] on qSOFA (quick sepsis related organ failure assessment score) combined with suPAR (soluble urokinase plasminogen activator receptor) for early risk detection and guidance of antibiotic treatment in emergency department. Early identification and initiation of antibiotics in sepsis is crucial but at times we fail to identify it leading to poor outcome of the patients. Earlier when qSOFA was used, sepsis was identified as qSOFA greater than 2 but in the article the authors have tried to identify sepsis when qSOFA is 1 combined with suPAR values. The approach is novel and we want to congratulate the authors for the same. The results shared for the antibiotic receiving group in the study also favors the approach over placebo. With these sweet parts there are some unclear sour aspects of the study too.

First, the surviving sepsis guidelines mentions that early initiation of antibiotic is necessary and that culture need to be taken prior to initiation of antibiotic. This is because a single dose of antibiotic can lead to negative culture reports despite infection. We could not find information on time of culture and pre-emptive administration of antibiotics in the study.

Second, the focus of study was qSOFA but in first part of the study recruitment of subjects was based on two signs of SIRS (Systemic inflammatory response syndrome). The criteria of qSOFA and SIRS is not interchangeable. No two parameters overlap between SIRS criteria (temperature, heart rate, respiratory rate and white blood cell count) and qSOFA parameters (respiratory rate, altered mental status and hypotension). Thus the recruitment likely included patient with only one common factor i.e. the respiratory rate, restricting recruitment to particular subset of patient and hence must have missed suitable patients which could fulfilled inclusion criteria based on blood pressure or neurological parameters. This affects generalizability of results. Hence also, the suPAR cut off values may be reflection of patients with respiratory pathology and likely also be the reason the meropenem group in the SUPERIOR (SUPar-guided doublE-blind randomized controlled trial of Initiation Of antibiotics foR presumed infection at the emergency department) study showed significant advantage compared to placebo, since respiratory cases constituting the majority of recruited patients.

Third and most importantly, the number needed to do an adequately powered study was supposed to be 110 in each group but only 91 patients in total were enrolled due to the covid pandemic which is less than 50% of the required sample size. The sample size is calculated based on statistical significance of primary objective. Though results were in favour of the Meropenem group but the numbers enrolled in study is statistically insufficient. Hence the inference from the study may be due to chance rather than fact. As such also the p value does not carry any significance in this study.

Fourthly, there might be ethical issue with the design. The placebo group receiving saline was devoid of beneficial effect of early initiation of antibiotic as all the patients recruited were treated as per sepsis protocol. This hold more significance especially when earlier studies have shown suPAR > 12 ng/mL independent predictor of unfavorable outcome or death in the first 28 days for patients with infection [2]. Is it ethical to deprive 1st dose in view of available literature?

Despite these, however the results of current study are motivating for a prospective study in future after taking into consideration the shortcomings observed in the current study.

## Data Availability

No datasets were generated or analysed during the current study.
